# Pharmacological Effects of *Centella asiatica* on Skin Diseases: Evidence and Possible Mechanisms

**DOI:** 10.1155/2021/5462633

**Published:** 2021-11-20

**Authors:** Kyoung Sik Park

**Affiliations:** Division of BT Convergence, College of Engineering, Cheongju University, Cheongju, Chungbuk, Republic of Korea

## Abstract

The medicinal herb *Centella asiatica* (L.) Urban known as gotu kola has been reported to exhibit a wide range of pharmacological activities. In particular, a significant body of scientific research exists on the therapeutic properties of preparations of *C. asiatica* or its triterpenes in the treatment of skin diseases. The present study is aimed to provide a comprehensive overview of the beneficial effects of *C. asiatica* on skin diseases. Peer-reviewed articles on the potent dermatological effects of *C. asiatica* were acquired from PubMed, Web of Science, Scopus, ScienceDirect, and SciFinder. This review provides an understanding of pharmacological studies which confirm the potent dermatological effects and underlying molecular mechanisms of *C. asiatica*. This medicinal plant and its triterpenes include asiaticoside, madecassoside, and their aglycones, asiatic acid and madecassic acid. These compounds exert therapeutic effects on dermatological diseases such as acne, burns, atopic dermatitis, and wounds via NF-*κ*B, TGF-*β*/Smad, MAPK, Wnt/*β*-catenin, and STAT signaling in *in vitro* and *in vivo* studies. However, additional rigorously controlled long-term clinical trials will be necessary to confirm the full potential of *C. asiatica* as a therapeutic agent.

## 1. Introduction

*Centella asiatica* (L.) Urban is a perennial plant that grows in swampy areas of tropical and subtropical regions of India, Southeast Asia, and Malaysia, as well as some temperate regions of China, Korea, Japan, and Taiwan [1]. The herb, also known as gotu kola or Indian pennywort, is a valued medicinal plant widely used in the Orient to treat infectious skin diseases and accelerate the healing of skin ulcers and wounds. In addition, internal preparations have been applied to treat dysentery, gastric ulcers, and syphilitic lesions [2].

Secondary metabolites found in the aerial parts of *C. asiatica* are classified into pentacyclic triterpenoids, sesquiterpenes, plant sterols, and saponins [3]. The herb is rich in pentacyclic triterpenoids (C_30_), of which the most abundant bioactive substances include asiaticoside, madecassoside, and their aglycones, asiatic acid and madecassic acid [1]. Essential oils from plants contain high levels of sesquiterpenes (C_15_) and monoterpenes (C_10_), including *α*-humulene, *ß*-caryophyllene, myrcene, bicyclogermacrene, and germacrene-D [4]. Other constituents found in the aerial part of *C. asiatica* have been characterized as chlorogenic acids, isomeric dicaffeolyl esters, and flavonoids such as catechin, epicatechin, kaempferol, and quercetin [5].

A growing body of research suggests therapeutic potentials of *C. asiatica* in treatment of neurological, endocrine, cardiovascular, digestive, respiratory, and dermatological diseases [6]. Investigations have reported that *C. asiatica* enhances the functions of the nervous system; *C. asiatica* and its triterpenes have a positive effect in relieving symptoms of Parkinson's disease [7] and Alzheimer's disease [8]. In addition, *C. asiatica* extracts have been reported to be effective in the treatment of endocrine diseases such as type 2 diabetes [9–11] and obesity [12]. Asiaticoside and asiatic acid, the active compounds of *C. asiatica,* have positive effects on cardiovascular diseases including hypertension [13, 14] and atherosclerosis [15, 16]. *C. asiatica* extract and its triterpenes exert protective effects against liver injury [17–20] and gastrointestinal tract damage [21, 22]. Asiaticoside and asiatic acid also have therapeutic effects on respiratory disorders such as pulmonary fibrosis [23], chronic obstructive pulmonary disease [24], and acute lung injury [25]. In particular, several *in vitro* and *in vivo* studies suggest that *C. asiatica* extracts and their triterpenes hold great promise as natural remedies that mitigate acne, burns, atopic dermatitis, and wounds [6, 26].

The aim of the current study was not only to review the pharmacological effects of *C. asiatica* on skin diseases but also to elucidate the possible underlying mechanisms of dermatological activity. Although a review paper had discussed the benefits of *C. asiatica* in dermatology a decade ago [26], it is necessary to include reports published after that time. Database searches using PubMed, Web of Science, Scopus, ScienceDirect, and SciFinder were performed until August 2021 to include up-to-date documented information in the present review. For data mining, the following descriptors were applied in the databases mentioned above: *C. asiatica*, triterpene, asiaticoside, madecassoside, asiatic acid, madecassic acid, therapeutic effect, treatment, prevention, skin disease, acne, burns, atopic dermatitis, and wounds. In almost all cases, original articles were obtained and the relevant data were extracted.

## 2. Pharmacological Effects of *Centella asiatica* on Skin Diseases

The therapeutic effect of *C. asiatica* on skin diseases has been reported to treat or relieve acne (Table 1), burns (Table 2), atopic dermatitis (Table 3), and wounds (Table 4) [6, 26].

### 2.1. Acne

Acne is a common chronic inflammatory skin disease that affects the pilosebaceous units of the skin [27]. The four main pathological factors involved in the development of acne include increased sebum production, irregular follicular desquamation, *Propionibacterium acnes* proliferation, and inflammation [28]. In particular, *P. acnes* proliferation may trigger the release of chemostatic factors such as neutrophils, which may cause follicular damage and rupture and leakage of bacteria, fatty acids, and lipids into the surrounding dermis. This process results in the formation of inflammatory lesions [29].

The methanol extract of *C. asiatica* was assayed for antibacterial activity using a disk diffusion assay [30]. The inhibition zones for the disks with 60 *μ*L of 15 mg/mL *C. asiatica* extract were <15 mm against *P. acnes*, indicating that the *C. asiatica* extract showed little antibacterial activity against *P. acnes*. Although the herbal mixture containing the *C. asiatica* extract exhibited high antimicrobial activity against *P. acnes* with 31.25 *μ*g/mL of minimum inhibitory concentration (MIC), it did not support the antibacterial activity against *P. acnes* [31]. Purified madecassoside is a major pentacyclic triterpene saponin from *C*. *asiatica*. In contrast to the low antimicrobial activity of *C. asiatica* extract against *P. acnes*, the purified madecassoside significantly inhibited the production of proinflammatory cytokine IL-1*β*, TLR2 expression, and nuclear translocation of NF-*κ*B in *P. acnes*-stimulated THP-1 human monocytic cells [32].

### 2.2. Burns

Burns result in the development of a dysregulated inflammatory and stress host response characterized by elevated levels of cytokines, chemokines, and acute phase proteins [33]. Following the inflammatory response, activation of keratinocytes and fibroblasts via various cytokines and growth factors helps restore vascular perfusion and further promotes wound healing. The next phase of healing involves wound remodeling, in which collagen and elastin are deposited and continuously transform fibroblasts into myofibroblasts. Over time, a delicate balance between contraction of myofibroblasts and reepithelialization determines the quality and pliability of the repaired wounds and determines the extent of scar formation, which is characterized by fibrous malposition of collagen fibers [34].

One study confirmed the burn wound-healing properties of asiaticoside and madecassoside [35]. Topical treatment with asiaticoside and madecassoside not only induced collagen synthesis, proliferation, and cell growth but also stimulated burn wound healing in male ICR mice. Cytol Centella® is a commercial cream formulated with a titrated extract of *C. asiatica*. Topical application of Cytol Centella® significantly stimulated burn wound contraction by facilitating collagen synthesis in male Wistar rats [36].

Topical application with Centiderm ointment made from *C. asiatica* ethanol extract significantly improved the objective (pliability, vascularity, pigmentation, height, and visual acuity scores) and subjective (dryness, itching, and irritation) signs in patients with second-degree burn wounds on their limbs [37]. In addition, the means of reepithelialization and complete healing were significantly better in the Centiderm group than in the control group. Polyester coated with herbal extract (5% *C. asiatica* extract and 2.5% *Aloe vera* extract) dressings on the burn wound area facilitated burn wound healing, reduced the sizes of burn wounds with higher % epithelialization, and decreased pain scores in another clinical trial [38].

### 2.3. Atopic Dermatitis

Atopic dermatitis (AD) is the most common inflammatory skin disorder that induces intense itching, edema, erythema, thickening, severe pruritus, and eczematous skin lesions. The pathogenesis of AD is multifactorial, involving immunologic processes including type 1 IgE dysfunction, defects in cell-mediated immune responses, and changes related to barrier dysfunction [39].

The therapeutic effect of a test material on AD is usually evaluated using phthalic anhydride (PA)-induced AD animal models. Titrated extract of *C. asiatica* (TECA) treatment attenuated the development of PA-induced AD by inhibiting the expression of iNOS and COX-2, NF-*κ*B activity, and the release of TNF-*α*, IL-1*β*, IL-6, and IgE [40]. Another similar report supports the therapeutic effect of *C. asiatica* on AD in PA-induced AD animal models. Topical treatment with *C. asiatica* phytosome inhibited the expression of iNOS and COX-2, the activity of NF-*κ*B, and the release of TNF-*α*, IL-1*β*, and IgE, leading to the suppression of inflammatory cell infiltration [41]. Furthermore, TECA mixed with astaxanthin augmented the inhibitory effect of TECA on PA-induced morphological changes in skin and ear thickness [42]. Interestingly, both topical and oral administration of *C. asiatica* ethanol extract decreased mast cell infiltration in the ear tissue and reduced the expression of various proinflammatory cytokines such as TNF-*α*, IL-4, IL-5, IL-6, IL-10, and IL-17 [43].

### 2.4. Wounds

Skin wounds are characterized by injury to the skin due to trauma, tears, cuts, or contusions. Wound healing is a physiological process that restores skin integrity and repairs the damaged tissues. Skin wound healing proceeds in four phases: hemostasis, inflammation, proliferation, and remodeling [44].

Asiaticoside was found to promote normal human skin cell migration, attachment, and growth in an *in vitro* wound healing model. ECa 233, a standardized extract of *C. asiatica*, induced keratinocyte migration and promoted wound healing through the activation of FAK, Akt, and MAPK signaling pathways [45].

Asiaticoside-rich hydrogel made from aerial parts of *C. asiatica* facilitated skin wound healing faster than the commercial cream and the untreated wounds in rabbits in an incision model [46]. The accelerated wound healing with *C. asiatica* hydrogel treatment may be owing to the formation of a thick epithelial layer, keratin, granulation tissues, fibroblasts, and collagen. The wound areas of rat skin treated with gelatin membranes containing *C. asiatica* methanol extract exhibited collagen deposition and a high number of capillaries, leading to enhanced skin wound healing [47].

In an *in vivo* excision wound model, there was significant wound healing in the animal group treated with topical spray containing *C. asiatica* methanol extract than in the untreated group [48]. Interestingly, asiaticoside nitric oxide gel promoted the healing rate of diabetic cutaneous ulcer wounds by regulating the Wnt/*β*-catenin signaling pathway and inhibiting the growth of bacteria on the wound surface in a rat wound model with diabetic cutaneous ulcers [49].

In contrast to the abundant *in vivo* evidence of the wound-healing effect of *C. asiatica*, clinical studies are sparse. Yet, one clinical trial showed a significant improvement in post-laser-resurfacing wound healing in patients with bilateral atrophic facial acne scars treated with ECa 233 gel for three months compared to the patients in the control group [50].

### 2.5. Other Skin Diseases

Vitiligo is a depigmenting skin disorder characterized by the selective loss of melanocytes which leads to pigment dilution in affected areas of the skin. It is the result of genetics and environmental causes in addition to metabolic stress, oxidative stress, and cell detachment abnormalities [51].

Madecassoside has attenuated mitochondrial damage caused by H_2_O_2_-induced oxidative stress in human epidermal melanocytes, suggesting that it could be a promising treatment for vitiligo mainly caused by oxidative stress (Table 5) [52].

Additionally, TECA promoted three-dimensional dermal papilla sphere formation by inhibiting the STAT activation in human dermal papilla cells, indicating TECA treatment may provide a useful strategy for promoting hair growth [53].

## 3. Underlying Mechanisms for the Pharmacological Effects of *Centella asiatica* on Skin Diseases

It has been reported that the feasible molecular mechanisms involved in the pharmacological effects of *C. asiatica* on skin diseases are as follows: NF-*κ*B, TGF-*β*/Smad, MAPK, Wnt/*β*-catenin, and STAT signaling.

### 3.1. NF-*κ*B Signaling

Nuclear factor-kappa B (NF-*κ*B) is a family of dimeric transcription factors that coordinate inflammatory responses, innate and adaptive immunity, and cellular differentiation, proliferation, and survival in almost all multicellular organisms [54]. The NF-*κ*B system is tightly regulated, and misregulation of NF-*κ*B has been implicated in a wide range of diseases ranging from cancer to inflammatory and immune disorders. As a result, the NF-*κ*B regulatory network and its dynamics offer a multitude of promising therapeutic targets that remain to be fully explored and translated into clinical use [55].

Titrated extract of *C. asiatica* (TECA) treatment suppressed NF-*κ*B activity and subsequently inhibited the expression of TNF-*α*, IL-1*β*, and IL-6 in a phthalic anhydride (PA)-induced atopic dermatitis (AD) animal model [40]. Furthermore, the addition of astaxanthin to TECA augmented the NF-*κ*B-mediated anti-inflammatory activity of TECA [42]. Another similar report supports the therapeutic effect of *C. asiatica* on AD via NF-*κ*B signaling. Topical treatment with *C. asiatica* phytosome inhibited the translocation of NF-*κ*B into the nucleus and the release of TNF-*α*, IL-1*β*, and IgE, leading to the suppression of inflammatory cell infiltration [41]. In an *in vitro* assay, purified madecassoside, a major pentacyclic triterpene from *C*. *asiatica*, significantly inhibited the nuclear translocation of NF-*κ*B and IL-1*β* production as well as TLR2 expression in *Propionibacterium acnes*-stimulated THP-1 human monocytic cells [32].

### 3.2. TGF-*β*/Smad Signaling

Transforming growth factor-*β* (TGF-*β*) is considered a crucial mediator in tissue fibrosis and causes tissue scarring largely by activating its downstream small mothers against decapentaplegic (Smad) signaling [56]. TGF-*β* plays a pivotal role in producing the myofibroblast phenotype, which is responsible for massive collagen deposition and contraction of wounds [57]. Since the TGF-*β*-induced Smad-dependent pathway is critical for the pathogenesis of all fibrotic diseases, various therapeutic strategies have been investigated to target the signaling pathway to attenuate aberrant skin scar formation [58].

Among the triterpenoid compounds of *C. asiatica*, the glycosides (asiaticoside and madecassoside) themselves, rather than their corresponding metabolites, asiatic acid and madecassic acid, are recognized as the main active constituents of *C. asiatica* herbs responsible for burn wound healing. Oral administration of both asiaticoside and madecassoside enhanced collagen type III synthesis by activating skin fibroblasts via the TGF-*β*/Smad pathway and facilitated burn wound healing in male ICR mice [59].

### 3.3. MAPK Signaling

Mitogen-activated protein kinase (MAPK) is an important signaling pathway in living beings in response to extracellular stimuli. There are five main subgroups manipulated by a set of sequential actions: ERK (ERK1/ERK2), c-Jun N (JNK/SAPK), *p*38 MAPK (*p*38*α*, *p*38*β*, *p*38*γ*, and *p*38*δ*), and ERK3/ERK4/ERK5 [60]. When stimulated, these groups have long been linked to multiple biological processes such as cell proliferation, differentiation, death, migration, invasion, and inflammation [61]. ECa 233, a standardized extract of *C. asiatica*, induced keratinocyte migration and subsequently promoted wound healing through the activation of FAK, Akt, and MAPK signaling pathways [45].

### 3.4. Wnt/*β*-Catenin Signaling

The Wnt/*β*-catenin pathway is one of the main processes in the regulation of a variety of biological processes including cell proliferation, apoptosis, and differentiation. It is also considered an important pathway in the healing of skin wounds. [62]. Asiaticoside nitric oxide gel promoted the healing rate of diabetic cutaneous ulcer wounds by regulating the Wnt/*β*-catenin signaling pathway and inhibiting the growth of bacteria on the wound surface in a rat model with diabetic cutaneous ulcers [49].

### 3.5. STAT Signaling

The signal transducer and activator of transcription (STAT) signaling pathway is a universally expressed intracellular signal transduction pathway and is involved in many crucial biological processes, including cell proliferation, differentiation, apoptosis, and immune regulation [63]. Hair growth can be induced from resting mouse hair follicles by topical application of Janus kinase (JAK) inhibitors, suggesting that JAK/STAT signaling is required for maintaining hair follicle stem cells in a quiescent state [64]. The titrated extract of *C. asiatica* (TECA) promoted three-dimensional dermal papilla sphere formation by inhibiting STAT activation in human dermal papilla cells, indicating that TECA treatment may provide a useful strategy for promoting hair growth [53].

## 4. Pharmacokinetic Properties of Active Compounds of *Centella asiatica*

The complex composition of *C. asiatica* renders pharmacokinetic studies of its multiple active compounds in humans or animal models particularly challenging. To date, pharmacokinetic studies on the active compounds of *C. asiatica* have primarily focused on *in vivo* absorption, distribution, metabolism, and elimination (ADME), as well as the bioavailability of *C. asiatica*-specific triterpenoids asiaticoside, madecassoside, and their aglycones asiatic acid and madecassic acid.

One pharmacokinetic study on asiatic acid was conducted after oral administration of an encapsulated water-soluble extract obtained from the aerial parts of *C. asiatica* in beagle dogs [65]. The main pharmacokinetic parameters of asiatic acid obtained from beagle dog plasma were *T*_1/2_, 4.29 h; *T*_max_, 2.70 h; *C*_max_, 0.74 *μ*g/ml; AUC_0–*t*_ and AUC_0–*∞*_, 3.74 and 3.82 *μ*g h/ml. Another pharmacokinetic study suggested that the absolute oral bioavailability of asiatic acid in rats is very low (16.25%), which may result from poor solubility and rapid metabolism [66]. Asiaticoside is converted *in vivo* to asiatic acid by hydrolytic cleavage of the sugar moiety, leading to little asiaticoside within the plasma after oral administration because of a proposed complete biotransformation into asiatic acid and its glucuronide and sulphate conjugates in a human study [67]. When pure madecassoside was administered orally as a single compound in rats, the pharmacokinetic parameters of madecassoside were *T*_max_ and *T*_1/2_ of 0.90 ± 0.14 h and 3.47 ± 0.68 h, respectively [68]. After a single oral dosing in rats, madecassoside was widely distributed in the heart, liver, spleen, lungs, and kidneys of rats and the levels of madecassoside in the liver and kidney were relatively higher than in other organs with primary elimination via the feces [69]. Notably, Anukunwithaya et al. conducted a disposition kinetic study on ECa 233, a standardized extract of *C. asiatica*, containing madecassoside (53.1%) and asiaticoside (32.3%) in rats [70]. Madecassoside and asiaticoside were rapidly absorbed, reaching maximum levels within 5–15 min after oral administration in rats, whereas madecassic and asiatic acids were found in negligible amounts. Both triterpenoid glycosides were extensively distributed in the brain, stomach, and skin within 1 h and remained there for at least 4 h after dosing. Interestingly, madecassoside and asiaticoside in ECa 233, administered orally at a dose of 100 mg/kg, were rapidly distributed to the skin with an AUC_(0–4)_ of 667.22 ± 121.06 and 114.50 ± 12.07 ng × h/g of skin tissue, respectively.

Few studies have examined the tissue distribution of triterpenes following oral administration or topical application, particularly the skin levels of these compounds. Although one study mentioned above [70] reported the dermal distribution of madecassoside and asiaticoside in a standardized extract of *C. asiatica* after oral dosing in rats, the cutaneous absorption rate of the active principles of topical application of *C. asiatica* has yet to be to be measured. Thus, further investigation is needed to understand the dermal distribution of these compounds and their metabolites.

## 5. Conclusions and Perspectives

Several *in vitro* and *in vivo* studies have demonstrated the therapeutic potential of *Centella asiatica* in the treatment of acne, burns, atopic dermatitis, and wounds. It has been suggested that the feasible molecular mechanisms involved in the pharmacological effects of *C. asiatica* on skin diseases include NF-*κ*B, TGF-*β*/Smad, MAPK, Wnt/*β*-catenin, and STAT signaling. However, further intensive clinical trials are required to confirm its efficacy as a therapeutic agent for treating skin diseases.

Medicinal products containing *C. asiatica* preparations are authorized and marketed in some of the European countries including Belgium, France, Greece, Italy, Portugal, and Spain. For external use, cutaneous cream 1% and powder 2% are recommended to support the local treatment of moderate or benign problems in wound formation and to aid in the local treatment of cutaneous ulcerations. In addition, oral tablets containing the titrated extract of *C. asiatica* (TECA) are authorized as potent wound-healing agents [71]. Since various formulations of *C. asiatica* have already been authorized and marketed as wound-healing therapeutics such as Madecassol®, Centellase®, and Blastoestimulina®, it could be considered that there is little need for clinical trials to confirm its effect on wound healing. However, extensive clinical studies are necessary to verify the healing effect of *C. asiatica* in subjects with various types and severities of wounds as well as the effect on skin diseases other than wounds, such as acne, burns, and atopic dermatitis.

## Figures and Tables

**Table 1:** Pharmaceutical effect of *C. asiatica* on acne.

*In vitro*				
Material tested	Cell line/assay system	Maximum concentration	Effect	Reference
*C. asiatica* methanol extract	Disk diffusion assay	15 mg/ml	Low antibacterial activity against *P. acnes*	[30]
Herbal mixture containing *C. asiatica* extract	Disk diffusion assay	ND	MIC for *P. acnes* = 31.25 *μ*g/ml	[31]
Purified madecassoside	*P. acnes*-stimulated THP-1 human monocytic cell line	500 *μ*M	TLR2 expression and nuclear translocation of NF-*κ*B ↓	[32]

ND: not determined; MIC: minimum inhibitory concentration.

**Table 2:** Pharmaceutical effect of *C. asiatica* on burns.

*In vivo*				
Material tested	Animal model	Dose, duration	Effect	Reference
Each of asiaticoside and madecassoside	Male SD rats	0.5 *μ*l on the area of burning wounds, 14 d	Collagen synthesis and cell proliferation ↑; burn wounds ↓	[35]
Cytol Centella® (titrated extract of *C. asiatica*)	Male Wistar rats	0.13 mg/mm^2^ on the area of burning wounds, 33 d	Burn wound contraction ↑; collagen synthesis ↑	[36]

*Clinical trial*				

Material tested	Study design/volunteer (*n*)	Dose, duration	Effect	Reference

Centiderm ointment containing *C. asiatica* ethanol extract	RCT, DB/patients with second-degree burn wounds on their limbs (*n* = 60)	Appropriate amounts on the area of burning wounds, 25 d	Objective and subjective signs ↑; mean of reepithelialization and healing completion ↑	[37]
Polyester coated with herbal extracts (5% *C. asiatica* extract and 2.5% *Aloe vera* extract)	RCT, DB/patients with second-degree burn wounds (*n* = 35)	Covering the area of burning wounds with the dressings with change every 3 days, 21 d	Burn wound healing ↑; sizes of burn wounds with higher % epithelialization ↓; pain scores ↓	[38]

RCT: randomized controlled trial; DB: double blind; objective: pliability, vascularity, pigmentation, height, and visual acuity score; subjective: dryness, itching, and irritation.

**Table 3:** Pharmaceutical effect of *C. asiatica* on atopic dermatitis.

*In vivo*				
Material tested	Animal model	Dose, duration	Effect	Reference
Titrated extract of *C. asiatica* (TECA)	Phthalic anhydride-induced AD model	40 or 80 *μ*g/cm^2^, 3 times a week for 4 wk	Development of AD ↓; hyperkeratosis and inflammatory cell infiltration ↓	[40]
*C. asiatica* phytosome	Phthalic anhydride-induced AD model	20 *μ*l/cm^2^, 3 times a week for 4 wk	Inflammatory cell infiltration ↓; expression of iNOS and COX-2 ↓; activity of NF-*κ*B and release of TNF-*α*, IL-1*β*, and IgE ↓	[41]
TECA and astaxanthin combination ointment	Phthalic anhydride-induced AD model	20 *μ*g/cm^2^, 3 times a week for 4 wk	Phthalic anhydride-induced skin morphological changes and ear thickness ↓	[42]
*C. asiatica* ethanol extract	2,4-Dinitrochlorobenzene-induced AD model	80 *μ*g/cm^2^ (topical) or 200 mg/kg/d (oral), 14 d	Mast cell infiltration ↓; expression of various cytokines ↓	[43]

**Table 4:** Pharmaceutical effect of *C. asiatica* on skin wounds.

*In vitro*				
Material tested	Cell line/assay system	Maximum concentration	Effect	Reference
Standardized extract of *C. asiatica* (ECa 233)	Human keratinocyte cell line (HaCaT)	100 *μ*g/ml	Cell migration ↑ wound healing activity ↑	[45]
*In vivo*				

Material tested	Animal model	Dose, duration	Effect	Reference
*C. asiatica* hydrogel	New Zealand white albino rabbits for an incision model	Appropriate amounts on the area of incisional wounds, 12 d	Wound healing ↑; formation of a thick epithelial layer, keratin, granulation tissues, fibroblasts, and collagen ↑	[46]
Gelatin membranes containing *C. asiatica* methanol extract	Male SD rats for an incision model	Covering the wound surfaces, 14 d	Wound healing ↑; collagen deposition and angiogenesis ↑	[47]
Topical spray containing *C. asiatica* methanol extract	Male Wistar rats for excision wound model	2.5 ml, once daily for 14 d	Wound healing ↑	[48]
Asiaticoside nitric oxide gel	Male SD rats for an incision model	0.2 ml, twice daily for 14 d	Healing rate of diabetic cutaneous ulcer wounds ↑; growth of bacteria in the wound surface ↓	[49]
*Clinical trial*				

Material tested	Study design/volunteer (*n*)	Dose, duration	Effect	Reference
Standardized extract of *C. asiatica* (ECa 233 gel)	RCT, DB/patients with bilateral atrophic facial acne scars (*n* = 30)	Appropriate amount on half-side of the face, twice daily for 3 mo	Post-laser-resurfacing wound healing ↑	[50]

RCT: randomized controlled trial; DB: double blind.

**Table 5:** Pharmaceutical effects of *C. asiatica* on other skin diseases.

*In vitro*				
Material tested	Cell line/assay system	Maximum concentration	Effect	Reference
Madecassoside	Human epidermal melanocytes	100 *μ*g/ml	Damage of mitochondria ↓; oxidative stress ↓	[52]
TECA	Human dermal papilla cells	25 *μ*g/ml	Potential of hair inductive capacity ↑	[53]

## Data Availability

The data used to support the findings of this study are available from the corresponding author upon request.
